# Dialysis-related thrombocytopenia: a case report

**DOI:** 10.1590/2175-8239-JBN-2020-0109

**Published:** 2021-02-26

**Authors:** Irene Faria Duayer, Maria Júlia Correia Lima Nepomuceno Araújo, Camila Hitomi Nihei, Maria Alice Fernandes Barcelos, Osni Braga, Zita Maria Leme Britto, Rosilene Mota Elias

**Affiliations:** 1 Hospital Nove de Julho, São Paulo, SP, Brasil.; 2 Universidade de São Paulo, Hospital das Clínicas, São Paulo, SP, Brasil.

**Keywords:** Blood Platelets, Blood Platelet Disorders, Dialysis, Platelet Activation, Extracorporeal Circulation, Plaquetas, Transtornos Plaquetários, Diálise, Ativação Plaquetária, Circulação Extracorpórea

## Abstract

Thrombocytopenia is frequently observed in hemodialysis patients, and its correct investigation and control remain a challenge. It is estimated that during the hemodialysis session there is a drop of up to 15% in the platelet count, with recovery after the end of treatment. This reduction in platelets is due to platelet adhesion and complement activation, regardless of the membrane material. Several studies with platelet surface markers demonstrate increased platelet activation and aggregation secondary to exposure to cardiopulmonary bypass. This case report describes a patient on hemodialysis who developed severe thrombocytopenia during hospitalization. Investigation and exclusion of the most common causes were carried out: heparin-related thrombocytopenia, adverse drug reaction, hypersplenism, and hematological diseases. Afterwards, the possibility of hemodialysis-related thrombocytopenia was raised, since the fall was accentuated during the sessions with partial recovery after the dialyzer change. Attention to the sterilization method and dialyzer reuse must be considered for correction. In the current case, reusing the dialyzer minimized the drop in platelet counts associated with hemodialysis.

## Resumo

Plaquetopenia é frequentemente observada em pacientes em hemodiálise, e sua correta investigação e controle permanecem um desafio. Estima-se que, durante a sessão de hemodiálise, ocorra queda de até 15% da contagem de plaquetas, com recuperação após o término do tratamento. Essa queda de plaquetas é decorrente de adesão plaquetária e ativação do complemento, independentemente do material da membrana. Vários estudos com marcadores de superfície plaquetária demonstram aumento da ativação e agregação plaquetária secundários à exposição à circulação extracorpórea. Este relato de caso mostra um paciente dialítico que evoluiu com plaquetopenia severa durante internação. Realizada investigação e exclusão de causas mais comuns: plaquetopenia relacionada à heparina, reação adversa a medicamentos, hiperesplenismo e doenças hematológicas, foi então aventada a possibilidade de plaquetopenia relacionada à hemodiálise após observação de que a queda se acentuava durante as sessões de hemodiálise com recuperação parcial após. Mudança do dialisador, atenção ao método de esterilização e realização do reuso devem ser consideradas para correção. No presente caso, a utilização do reuso minimizou a plaquetopenia associada a hemodiálise.

## Introduction

Renal replacement therapy has improved in recent decades, particularly with the use of synthetic and highly biocompatible dialyzer membranes. During hemodialysis treatment, patients are exposed to a variety of components of the dialysis circuit and thrombocytopenia is not uncommon^1^.

The platelets are fragments of cytoplasm that are derived from the megakaryocytes. After being released from the bone marrow, they are sequestered in the spleen for 24 to 48 hours. The spleen contains about 30% of the circulating platelets, whose lifespan is approximately 7 days. Platelets are removed from the bloodstream by macrophages^2^. Platelet number under normal conditions range from 150,000 to 450,000/mm^3^ in peripheral blood^3^. 

Decreased platelet count occurs due to reduced bone marrow production, increased platelet destruction, and other causes such as drugs or alcohol consumption. Usually, a myelogram helps to classify the origin of the thrombocytopenia. The increase in the number of megakaryocytes indicates their destruction or consumption and the decrease indicates lower production^2^^,^ 3.

It is estimated that a hemodialysis session can cause a decrease in platelet count by 15%, with recovery after the end of the treatment^1^. This is due to adhesion and complement activation, regardless of membrane material^4^^,^ 5.

This case report is of a patient who developed dialysis-related thrombocytopenia with improvement after the reuse of the dialyzer.

## Case report

**Figure 1:**
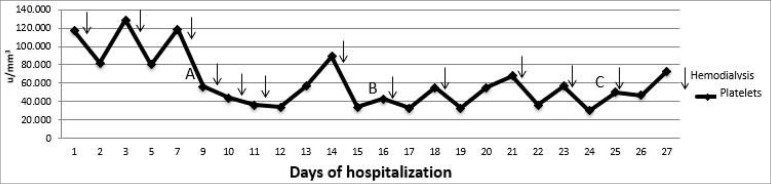
Evolution of platelet counts during hospitalization. Arrows indicate hemodialysis sessions. A: Heparin suspension; B: Switch to Fresenius Helixone Fx60 filter; C: Switch to Fresenius Hemoflow filter.

A 70-year-old man in stable condition on hemodialysis for 5 months was admitted to the hospital with a malfunctioning arteriovenous fistula. He had a medical history of hypertension, diabetes, unilateral nephrectomy due to nephrolithiasis, cured prostate cancer 5 years earlier, and end-stage renal disease secondary to diabetic kidney disease. He denied current smoking or alcohol use. He had 3 hemodialysis sessions per week using a Fresenius Hemoflow 80 Capilar, reused according to Brazilian legislation. He was taking the following medications: glargine insulin, hydralazine, valsartan, gabapentin, simvastatin, pantoprazole, sevelamer, alprazolam, ezetimibe, oxybutynin, erythropoietin, calcium citrate, and cholecalciferol. At admission his platelet count was 105,000/mm^3^ (Reference value - RV: 150,000 - 450,000/mm^3^). Angiography and angioplasty of the fistula were performed with no success and a long-term hemodialysis catheter was implanted. After subsequent hemodialysis sessions (Fresenius Helixone Fx 800 dialyzer, single use) platelet counts drop to 14.000/mm^3^. The patient remained asymptomatic during sessions with no bleeding. Thrombocytopenia was confirmed by manual counting, excluding the presence of clots and no signs of thrombotic microangiopathy. The oscillatory pattern of thrombocytopenia was noteworthy, as shown in Figure 1. 

The calculated 4T score^6^ suggested an intermediate probability of heparin-induced thrombocytopenia (HIT), and therefore heparin products were discontinued, including the one used to seal the catheter. Pantoprazole was replaced by ranitidine. As there was no improvement, the hypothesis of thrombocytopenia related to the capillary membrane was raised, and the dialyzer was switched to a Fresenius Helison Fx 60. Nevertheless, the patient remained with low platelets, reaching 16.000/mm^3^. An hematological evaluation was requested, but a medullar cause was ruled out after an extensive investigation. Serum protein electrophoresis, serum immunoelectrophoresis, light chain measurement, and hepatitis and HIV serology were all normal. Normocellular myelogram and bone marrow biopsy were with no anomalous immunophenotype population. Liver laboratory tests were normal with no signs of liver disease or splenomegaly in ultrasound. The patient presented with oscillatory thrombocytopenia, with no improvement from heparin suspension, normal platelets production, absence of hypersplenism, and a marked drop in platelets during the hemodialysis session (Figure 1). At the time, he presented normal coagulogram and HDL, absence of leukopenia, elevated d-dimer, and slightly increased fibrinogen (values: TP INR 1.0 - 12.9 sec - RV: 0.8-1.2; TTPA R 1.06 - 35.5 sec - RV: < 1.25; DHL 142 IU / L - RV 85-227 IU/L; fibrinogen 424 mg/dL - RV: 200-400 mg/dL; D-Dimer 1.12 µg/mL - RV: < 0.5 µg/mL). No complement or anti-heparin-PF4 antibodies dosage was performed. 

**Figure 2:**
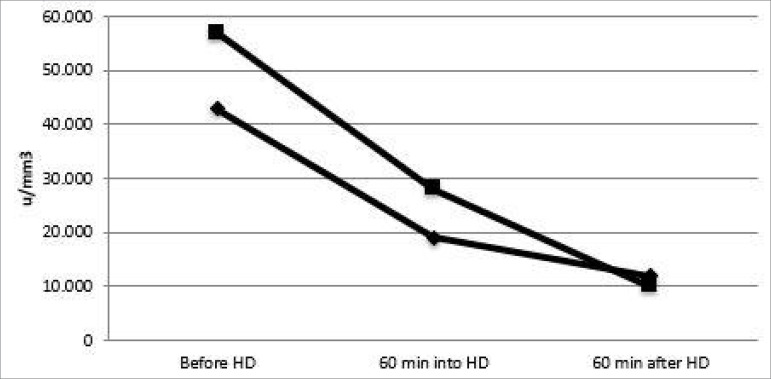
Platelet counts evolution during 2 hemodialysis sessions. HD, hemodialysis; min, minutes.

To investigate the dialysis effect, we measured platelet levels immediately before dialysis, after 60 minutes of the beginning, and 60 minutes after the end of treatment. The patient exhibited a decrease in platelet count above the expected 15%^1^. Pre-dialysis platelet count was 43,000/µL, 1 hour after it was 19,000/mm^3^, and 1 hour after the end of the session, 12,000/mm^3^ (Figure 2). 

Since the patient's bone marrow was normal and he had platelet consumption concomitant with the dialysis session (Figure 1), the dialyzer was switched from Fresenius Helixone Fx 60 to Fresenius Hemoflow, the same used in the outpatient clinic. The same pattern of platelet consumption was observed. Pre-dialysis platelet count was 57,000/mm^3^, 60 minutes into dialysis it was 28,000/mm^3^, and 60 minutes after the end it was 10,000/mm^3^ (Figure 2) 

Since there was no sign of bleeding and platelet increased to 90,000/mm^3^, the patient was discharged to continue ambulatory evaluation. After 3 sessions of hemodialysis in the satellite clinic using a dialyzer Fresenius Hemoflow without heparin, the patient had a pre-dialysis platelet count of 81,000/ mm^3^ and a fall to 77.000/mm^3^ during the session, which was within the expected 15%. One and two months after discharge, the platelet dosage in the clinic routine was 171,000/ mm^3^ and 151,000/ mm^3^. Hemodialysis heparin was then restarted and after 3 months the platelet count was 157,000/ mm^3^ when he was last seen by a hematologist. 

## Discussion

Thrombocytopenia is often observed in patients on renal replacement therapy and its investigation and treatment remain a challenge. After excluding bone marrow diseases, immune or non-immune conditions that increase destruction, heparin related, adverse drug reaction and hypersplenism, platelet consumption associated with hemodialysis session is the main hypothesis. In hemodialysis patients, platelet number tends to be reduced, in the range of 175-180.000/mm^3^ compared with 250.000/mm^3^ in healthy controls. The study reports a decrease of 5-15% of platelets in the first half-hour of dialysis with a recovery at the end of the session, and no reports of adverse bleeding events. The dialyzer material, its sterilization method, and its reuse interfere with this platelet drop^1^. 

The dialyzer can be rinsed after use for blood removal, chemically cleaned, sterilized, and reused. The reuse of the dialyzer is a safe and effective practice, employed worldwide. In the 1990s, 80% of hemodialysis patients in the United States reused the dialyzer, but this practice has been decreasing over the years. As for disadvantages, this technique presents a potential for dialysis contamination by bacteria and endotoxins during the reprocessing process, patient and staff exposure to chemical substances, and potential loss of b2-microglobulin clearance. As advantages, it reduces exposure to industrial residual chemicals used in the manufacture of new dialyzers and increases dialyzer biocompatibility since there is reduced activation of the immune system^7^.

Nowadays, cellulose dialyzers have been replaced by dialyzers that generate less activation of the complement system, being more biocompatible, such as those based on cellulose acetate, polysulfone, and polymethacrylate, as used in this case. Additionally, complement-independent platelet activation pathways are being studied as mechanisms of thrombocytopenia^8^^,^ 9.

Changes in blood flow rate, turbulent blood flow, increased hematocrit secondary to ultrafiltration, and exposure of blood components to the dialyzer material may contribute to hypercoagulability in extracorporeal circuits. Activation via direct contact is postulated as the main triggering factor. Activation of the coagulation cascade and thrombin generation contributes to platelet consumption, as thrombin directly activates platelets and consumes platelets in the formed clot. Associated with this, platelet activation resulting from adhesion to the dialyzer membrane may per se activate the coagulation cascade and contribute to platelet consumption^5^. The slightly elevated levels of the d-dimer presented by the patient may suggest the activation of this pathway and platelet consumption more likely than the classical complement pathway, once the patient had no other factor such as leukopenia and no cellulose-based dialyzer was used. Unfortunately, in this case, no complement was dosed.

Reports in the literature are consistent with the findings observed in this case. There are at least^75,^ 8 ^,^ 10 ^-^ 14 reports of thrombocytopenia secondary to dialysis even with the use of biocompatible dialyzer membranes, and considerations about triggering mechanisms of platelet consumption beyond the complement pathway are postulated^15^. Distinct strategies for its treatment were adopted, ranging from change of the dialysis material, change of sterilization method, or washing the system with saline prior to therapy^1^. Unfortunately, we have no information on antibodies dosage for proper exclusion of HIT, although the platelet reduction suggested a hemodialysis session-related cause and there was no improvement after heparin suspension. This report shows, for the first time, that the dialyzer reuse strategy could maintain a safe and effective hemodialysis.

The sterilization method varies according to each manufacturer, and each membrane has a direct effect on biocompatibility. Sterilization can be made by electron beam or gamma rays. In a cohort study of 1,706 patients in Canada, the use of electron beam-sterilized dialyzer was associated with significant thrombocytopenia following dialysis. Electron beam sterilization can probably alter membrane properties, making it less biocompatible and its effects are still being studied^14^.

Several studies with platelet surface markers show increased platelet activation and aggregation when exposed to extracorporeal circuit materials, even with the use of biocompatible membranes. The fall in platelet number is expected and transient at the beginning of the dialysis. Greater attention should be given to patients who persist with low levels. Change of dialyzer material, attention to the sterilization method, and dialyzer reuse practice should be considered to try to resolve the adverse event^9^. In the case of our patient, changing the dialyzer was not enough, and the reuse of the dialyzer was necessary.
